# Estrogen administration modulates hippocampal GABAergic subpopulations in the hippocampus of trimethyltin-treated rats

**DOI:** 10.3389/fncel.2015.00433

**Published:** 2015-11-05

**Authors:** Valentina Corvino, Valentina Di Maria, Elisa Marchese, Wanda Lattanzi, Filippo Biamonte, Fabrizio Michetti, Maria Concetta Geloso

**Affiliations:** ^1^Institute of Anatomy and Cell Biology, Università Cattolica del Sacro CuoreRome, Italy; ^2^Institute of Histology and Embryology, Università Cattolica del Sacro CuoreRome, Italy

**Keywords:** trimethyltin, estrogen, hippocampal neurodegeneration, glutamic acid decarboxylase, parvalbumin, neuropeptide Y (NPY)

## Abstract

Given the well-documented involvement of estrogens in the modulation of hippocampal functions in both physiological and pathological conditions, the present study investigates the effects of 17-beta estradiol (E2) administration in the rat model of hippocampal neurodegeneration induced by trimethyltin (TMT) administration (8 mg/kg), characterized by loss of pyramidal neurons in CA1, CA3/hilus hippocampal subfields, associated with astroglial and microglial activation, seizures and cognitive impairment. After TMT/saline treatment, ovariectomized animals received two doses of E2 (0.2 mg/kg intra-peritoneal) or vehicle, and were sacrificed 48 h or 7 days after TMT-treatment. Our results indicate that in TMT-treated animals E2 administration induces the early (48 h) upregulation of genes involved in neuroprotection and synaptogenesis, namely *Bcl2, trkB, cadherin 2* and *cyclin-dependent-kinase-5*. Increased expression levels of *glutamic acid decarboxylase* (*gad*) *67, neuropeptide Y (Npy*), *parvalbumin, Pgc-1α* and *Sirtuin 1* genes, the latter involved in parvalbumin (PV) synthesis, were also evident. Unbiased stereology performed on rats sacrificed 7 days after TMT treatment showed that although E2 does not significantly influence the extent of TMT-induced neuronal death, significantly enhances the TMT-induced modulation of GABAergic interneuron population size in selected hippocampal subfields. In particular, E2 administration causes, in TMT-treated rats, a significant increase in the number of GAD67-expressing interneurons in CA1 stratum oriens, CA3 pyramidal layer, hilus and dentate gyrus, accompanied by a parallel increase in NPY-expressing cells, essentially in the same regions, and of PV-positive cells in CA1 pyramidal layer. The present results add information concerning the role of *in vivo* E2 administration on mechanisms involved in cellular plasticity in the adult brain.

## Introduction

Many findings support the modulatory role of estrogens, whose effects are mediated mainly by 17-beta estradiol (E2), on brain functions, in particular at the hippocampal level (Spencer et al., 2008), where they are responsible for the enhancement of glutamate transmission, the induction of long-term potentiation and the modulation of inhibitory activity (Brann et al., 2007; Spencer et al., 2008). In addition to their well-documented neuroregulatory effects, both epidemiological observations in humans and experimental data support the efficacy of estrogens as neuroprotective agents in a variety of neurologic diseases, including neurodegenerative diseases (for review, see Garcia-Segura et al., 2001; Amantea et al., 2005; Brann et al., 2007; Arevalo et al., 2015), ischemia (Dai et al., 2007; Inagaki and Etgen, 2013), and experimental models of temporal lobe epilepsy (Azcoitia et al., 1998, 1999a,b; Picazo et al., 2003; Melcangi and Panzica, 2006; Velísková, 2006; Velisek et al., 2013). E2 exerts neuroprotection through multiple mechanisms, including the enhancement of antiapoptotic and/or anti-inflammatory pathways and the modulation of neuronal plasticity (Amantea et al., 2005; Brann et al., 2007). The latter includes the E2-mediated regulation of dendritic spine formation and density and/or modulation of the excitatory/inhibitory synaptic balance (Brinton, 2009).

In this respect, special attention has been focused on the effects mediated by E2 administration on different GABAergic hippocampal subpopulations (Spencer et al., 2008), namely neuropeptide Y (NPY) (Nakamura and McEwen, 2005; Velísková and Velísek, 2007; Ledoux et al., 2009) and parvalbumin (PV)-expressing cells (Sotonyi et al., 2010; Koh, 2014). NPY-positive interneurons are known to play a relevant role in the inhibition of hippocampal circuitry, where they modulate excitatory neurotransmission, regulate hyperexcitability (Baraban et al., 1997; Vezzani et al., 1999), and are also involved in the modulation of dentate neurogenesis (Gray, 2008; Decressac and Barker, 2012; Geloso et al., 2015). PV-expressing interneurons play a crucial role in the functional properties of the hippocampus: they participate in the synchronization of oscillations in the hippocampal network (Klausberger et al., 2005; Donato et al., 2013), and their functional/structural impairment has been associated with severe neurologic disorders, including autism (Lawrence et al., 2010; Cellot and Cherubini, 2014), schizophrenia (Cabungcal et al., 2013; Jiang et al., 2013), epilepsy (Andrioli et al., 2007), and Huntington’s disease (Cicchetti et al., 2000).

The trimethyltin (TMT)-induced model of hippocampal neurodegeneration is suitable not only to study the neuronal and glial responses that accompany progressive neuronal death and the signaling pathways associated with neuronal damage (Balaban et al., 1988; Koczyk, 1996; Geloso et al., 2011; Corvino et al., 2013; Lattanzi et al., 2013), but also to investigate possible neuroprotective strategies (for review, see Shin et al., 2011; Corvino et al., 2013). In rats, a single injection of TMT causes progressive neuronal death of CA1 and CA3/hilus pyramidal cells, developing over 3 weeks (Balaban et al., 1988; Koczyk, 1996; Geloso et al., 2011; Corvino et al., 2013; Lattanzi et al., 2013) and is associated with selective sparing of the GABAergic subpopulations expressing PV and calretinin (Geloso et al., 1996, 1997, 1998), astroglial (Geloso et al., 2004; Pompili et al., 2004; Latini et al., 2010) and microglial activation (Brabeck et al., 2002), enhanced neurogenesis (Corvino et al., 2005), seizures and cognitive impairment (for review, see Koczyk, 1996; Geloso et al., 2011). Experimental findings suggest that it may also be useful to study the interplay between neuronal death and the functional impairment of neurotransmission (Koczyk, 1996; Ishida et al., 1997; Kruger et al., 2005), as well as to examine cellular and molecular events involved in hippocampal plasticity (Koczyk, 1996).

The impairment of glutamatergic neurotransmission (Koczyk, 1996; Geloso et al., 2011) and changes in the GABAergic system (Dyer and Boyes, 1984; Nishimura et al., 2001; Kruger et al., 2005) have been reported. The latter include an increased expression of glutamic acid decarboxylase (GAD) 65 and GAD67, the rate-limiting enzymes in GABA synthesis, in the early phase of neuronal damage (Nishimura et al., 2001) and the modulation of interneuron subpopulations expressing NPY and somatostatin (Sadamatsu et al., 1998; Ishikura et al., 2002).

The present study was designed to investigate the effects of E2 administration in the experimental model of TMT-induced hippocampal neurodegeneration, and to evaluate its effects on neuronal death and interneuron reorganization, with the aim of adding information concerning the role of *in vivo* E2 administration as a possible neuroprotective approach.

## Materials and Methods

### Animal Treatment and Experimental Design

Two-month-old female Wistar rats (200–250 g) were bilaterally ovariectomized under ketamine (75 mg/Kg)/medetomidine hydrochloride (0.5 mg/Kg, intramuscular) anesthesia. Ovariectomy was preceded by a midline dorsal skin incision, approximately halfway between the middle of the back and the base of the tail, as described by other groups (Khajuria et al., 2012). Rats were then housed for 3 weeks in order to eliminate endogenous plasma estradiol. They then received a single intra-peritoneal (i.p.) injection of TMT chloride (Sigma, St Louis, MO, USA) dissolved in saline at a dose of 8 mg/Kg body weight in a volume of 1 ml/kg body weight, as previously described (Geloso et al., 1996, 1997, 2011). A CTRL group received the same volume of saline.

As much evidence supports the notion that estrogen administration shows beneficial effects when delivered as a pretreatment (Henderson, 1997; Yune et al., 2004; Brann et al., 2007; Samantaray et al., 2010; Velisek et al., 2013), we administered E2 in two doses (days 1 and 2 after TMT injection) in the time frame between the initiating event (TMT injection) and the occurrence of the first TMT-induced structural/functional hippocampal changes. We chose this approach in order to counteract early events involved in TMT-induced hippocampal injury, which are known to be delayed and to become apparent from post-intoxication day 2 (Ishikura et al., 2002; Geloso et al., 2011).

One hour after TMT injection (post-treatment day 0) the rats were divided into four experimental groups (CTRL + oil, CTRL + E2, TMT + oil, TMT + E2) and received E2 or vehicle (sesame oil) treatment. E2-3 benzoate (Sigma, St Louis, MO, USA) was administered at a dose of 0.2 mg/kg i.p. in accordance with the neuroprotective effects reported in previous studies performed by other groups in different models of brain injury (Azcoitia et al., 1999b; Picazo et al., 2003; Gresack and Frick, 2006; Twining et al., 2013). The same E2/vehicle dose was administered on post-treatment day 1. After treatment the animals were returned to their cages and housed on a 12 h light/dark cycle with free access to food and water.

Rats were sacrificed at two time points after treatment: 48 h after TMT/saline administration (T1, i.e., 24 h after the last E2 administration), to explore early molecular events related to E2 treatment, and 7 days after TMT/saline injection (T2, i.e., 6 days after the last E2 administration), when neuronal loss induced by TMT injection is clearly detectable by histological analysis (Latini et al., 2010; Corvino et al., 2011), to explore late effects of E2 treatment.

At time point T1, E2 serum levels were checked. Heart blood was collected from deeply anesthetized animals (ketamine/diazepam 1:1 i.p.) immediately before they were sacrificed, via cardiac puncture, and processed to obtain serum (Leuner et al., 2004). Serum E2 levels were detected by Chemiluminescent Microparticle Immunoassay (CMIA; Abbott Laboratories, Longford, Ireland) of duplicate samples as per the manufacturer’s protocol. Assays showed significantly higher E2 levels in E2-treated rats (mean plasma estradiol levels in E2-treated rats = 160 ± 76,43 pg/ml) compared with vehicle-injected animals (mean plasma estradiol levels in oil treated rats = 16,33 ± 2,9 pg/ml; Mann–Whitney test *p* < 0.05) (Gresack and Frick, 2006; Twining et al., 2013).

All animal procedures were approved by the Ethics Committee of the Catholic University and were fully compliant with the Italian Ministry of Health guidelines (Legislative Decree No. 116/1992) and European Union (Directive No. 86/609/EEC) legislation on animal research. Efforts were made to limit the number of animals used and to minimize their suffering. ARRIVE guidelines were followed.

### Gene Expression Analysis

Animals intended for gene expression analysis were sacrificed by decapitation after deep anesthesia (ketamine/diazepam 1:1 i.p.) 48 h after TMT or saline treatment (T1; CTRL + vehicle: *n* = 4, CTRL + E2: *n* = 4, TMT + vehicle: *n* = 4, TMT + E2: *n* = 4). The hippocampi were removed bilaterally and processed for total RNA isolation, reverse transcription (RT)-PCR and quantitative real time PCR (qPCR), as previously described (Corvino et al., 2012, 2014). The following genes where amplified using sequence-specific oligonucleotide primers (Supplementary Table S1): *B-cell CLL/lymphoma 2* (*Bcl2*), *brain-derived neurotrophic factor* (*Bdnf*), *cadherin 2* (*Cdh2*), *cyclin-dependent kinase 5* (*Cdk5*), *glutamate decarboxylase 1* (*Gad1* also known as *Gad67*), *neurotrophic tyrosine kinase, receptor, type 2* (*Ntrk2* also known as *trkB*), *neuropeptide Y* (*Npy*), *parvalbumin* (*Pva*), *peroxisome proliferator-activated receptor gamma coactivator 1 alpha* (*Pgc-1*α, encoded by *Ppargc1a*) and *sirtuin 1* (*Sirt1*); *cytochrome P450 19* or *aromatase* (*Cyp19a1)*.

The 2^-ΔΔCt^ method (Livak and Schmittgen, 2001) was applied to calculate fold changes (FC) in gene expression, using the gene encoding the glyceraldehyde-3-phosphate dehydrogenase (*Gapdh*) as the housekeeping reference for data normalization, as already described (Corvino et al., 2012, 2014).

### Immunocytochemistry

Rats from the four experimental groups intended for histology and immunocytochemistry were sacrificed at time point T2. Under deep anesthesia (ketamine/diazepam 1:1 i.p.), the animals were perfused with 4% phosphate-buffered saline (PBS) paraformaldehyde, the brains were removed from the skull and 40 μm serial sagittal sections, from 0.9 to 3.4 mm lateral to the midline, according to Paxinos and Watson (1986) atlas, were collected in PBS. Every sixth section was processed for Nissl or Fluoro Jade C (Chemicon, Temecula, CA, USA) staining to detect neuronal death, or stained for immunocytochemistry with anti-GAD67, -PV, and -NPY antibodies to study their expression in different interneuronal subpopulations. Sections were incubated overnight with mouse monoclonal anti-GAD67 (Millipore, Temecula, CA, USA; 1:2000), mouse monoclonal anti-PV (Swant, Bellinzona, Switzerland; 1:10000), rabbit polyclonal anti-NPY (AbCam, Cambridge, UK; 1:2000) antibodies. The reaction was developed using the avidin–biotin peroxidase complex (ABC method, Vector Burlingame, CA). 3,3′-diaminobenzidine (Sigma, St. Louis, MO, USA) was used as a chromogen.

Co-expression of the GABAergic interneuron marker GAD67 (Freund and Buzsáki, 1996; Rudy et al., 2011) and PV or NPY was identified by fluorescent double-labeling using mouse monoclonal anti-GAD67, rabbit polyclonal anti-PV (AbCam, Cambridge, UK; 1:2000) or rabbit polyclonal anti-NPY antibody, revealed using secondary cyanine-3-conjugated antibody (donkey anti-mouse Cy3, 1:400, 1 h at room temperature (Jackson Immunoresearch Laboratories, West Grove, PA, USA), or secondary FITC-conjugated antibody (goat anti-rabbit FITC, Vector, Burlingame, CA, USA 1:200, 1 h at room temperature). Controls were prepared by omitting the primary antibody. Co-localization of the different markers was examined with a LSM 510 META confocal laser scanning microscopy system (Zeiss, Oberkochen, Germany).

### Quantitative Analysis

#### Stereological Estimations

The optical fractionator stereological design (West et al., 1991) was used to obtain unbiased estimates of total Nissl-stained, Fluoro Jade C-stained, GAD67-, PV-, or NPY-immunoreactive (IR) neurons in the regions of interest, using the Stereo Investigator system (Stereo Investigator software, Version 9, MicroBrightField Europe, Magdeburg, Germany), essentially as previously described (Corvino et al., 2012). A stack of MAC 6000 controller modules (MBF Bioscience, Williston, VT, USA) was configured to interface with a Nikon Eclipse 80i microscope with a motorized stage and a digital color camera (MBF Bioscience q imaging) with a Pentium II PC workstation.

To detect the extent of TMT-induced neuronal death, Nissl-stained neurons located in the CA1 and CA3 pyramidal layer and in the hilus, which are the main sites of TMT-induced neuronal loss (Geloso et al., 2011), were counted. Only cells showing unambiguous neuronal morphology, with regularly shaped nuclei showing clearly detectable nucleoli and with no signs of nuclear fragmentation were counted in the hippocampi of the four experimental groups (CTRL + oil *n* = 5; CTRL + E2 *n* = 6; TMT + oil *n* = 8; TMT + E2 *n* = 8). A three-dimensional optical dissector counting probe (*x, y, z* dimension of 30 μm × 30 μm × 10 μm, respectively) was applied to a systematic random sample of sites in the region of interest at a magnification of 100×. Quantitative analysis of Fluoro Jade-C positive degenerating neurons was performed in the same conditions in the CA1 and CA3 subfields.

GAD67-IR interneurons were counted in the stratum oriens, radiatum and pyramidal layer of the CA1 and CA3 subfields, in the hilus and in the dentate gyrus (DG) (counting probe: *x, y, z* dimension of 200 μm × 200 μm × 10 μm, respectively, magnification of 40×; CTRL + oil *n* = 6, CTRL + E2 *n* = 5, TMT + oil *n* = 5, TMT + E2 *n* = 6).

The quantification of PV- and NPY-IR neurons was restricted to those hippocampal layers containing a higher cell density of IR cells, since the stereological approach requires that in each sampling area 1–2 cells should be counted on average. Accordingly, PV-IR interneurons were counted only in the stratum oriens and in the pyramidal layer of the CA1 and CA3 subfields and in the granular layer of the DG (Andressen et al., 1993). A three-dimensional optical dissector counting probe (counting probe: *x, y, z* dimension of 200 μm × 200 μm × 10 μm, respectively) was applied to a systematic random sample of sites in the region of interest at a magnification of 40× (CTRL + oil *n* = 8, CTRL + E2 *n* = 7, TMT + oil *n* = 9, TMT + E2 *n* = 9).

NPY-IR interneurons were counted in the stratum oriens and pyramidal layer of the CA1 subfield and in the hilus, which are the hippocampal regions exhibiting the highest cell density, in line with previous observations (Deller and Leranth, 1990; Sperk et al., 2007), (counting probe: *x, y, z* dimension of 200 μm × 200 μm × 10 μm, respectively, magnification of 40×; CTRL + oil: *n* = 5, CTRL + E2: *n* = 5, TMT + oil: *n* = 7, TMT + E2: *n* = 7).

#### Confocal Microscope Double-staining Quantitative Analysis

Double-stained PV/GAD67 (CTRL + vehicle: *n* = 4, CTRL + E2: *n* = 4, TMT + vehicle: *n* = 4, TMT + E2: *n* = 4) or NPY/GAD67 (CTRL + vehicle: *n* = 3, CTRL + E2: *n* = 3, TMT + vehicle: *n* = 4, TMT + E2: *n* = 4) interneurons were quantified in the four experimental groups using z-scan confocal microscopy at 40× magnification. The entire length of the above regions of interest (CA1 stratum oriens, CA1 pyramidal layer, CA3 stratum oriens, CA3 pyramidal layer, and hilus) was evaluated through the septo-temporal axis of the hippocampus in 1-in-12 series of sections, as previously described (Corvino et al., 2012, 2014). Analysis of co-localization of markers was performed on well-stained cells with clearly visible neuronal bodies. The number of double-labeled cells was counted manually by an experimenter who was unaware of the group assignment. Estimates of the total number of cells positive for each marker were obtained using the following formula: *E* = *k*∑ *N*, where *E* is the estimate of the total number of stained cells in each case, ∑ *N* is the sum of *n*-values in the *n* sections considered, and *k* indicates that every kth section was considered (*k* = 12). *N* was corrected according to Abercrombie’s formula: *N* = *n t*/(*t* + *D*), where *n* is the number of cells counted in each section, *t* is the section thickness, and *D* is the mean diameter of the cells (Abercrombie, 1946).

The quantification of double-stained cells was expressed as the percentage of PV/GAD67 or NPY/GAD67 double-labeled cells in relation to the total number of PV-IR or NPY-IR cells.

#### Statistical Analysis

Three-way Repeated-Measures (RM) ANOVA with TMT and E2 treatments as the between-subjects factors and hippocampal subfields as the within-subjects factor or two-way ANOVA with TMT and E2 treatment as main factors were performed to analyze statistically significant differences between the groups, as previously described (Geloso et al., 1996, 1997, 1998). When appropriate, *post hoc* comparisons were made using Tukey’s HSD test, with a significance level of *p* < 0.05. Results are expressed as mean ± SE.

In order to assess the statistical significance of the gene expression changes for each gene in each experimental group, an unpaired *t*-test was used to compare the ΔCt-values across the replicates, setting the *p*-value cut-off at 0.05, as previously described (Corvino et al., 2012, 2014). Comparisons were made across all four experimental groups.

## Results

### Early Molecular Events Induced by E2 Administration

**FIGURE 1:**
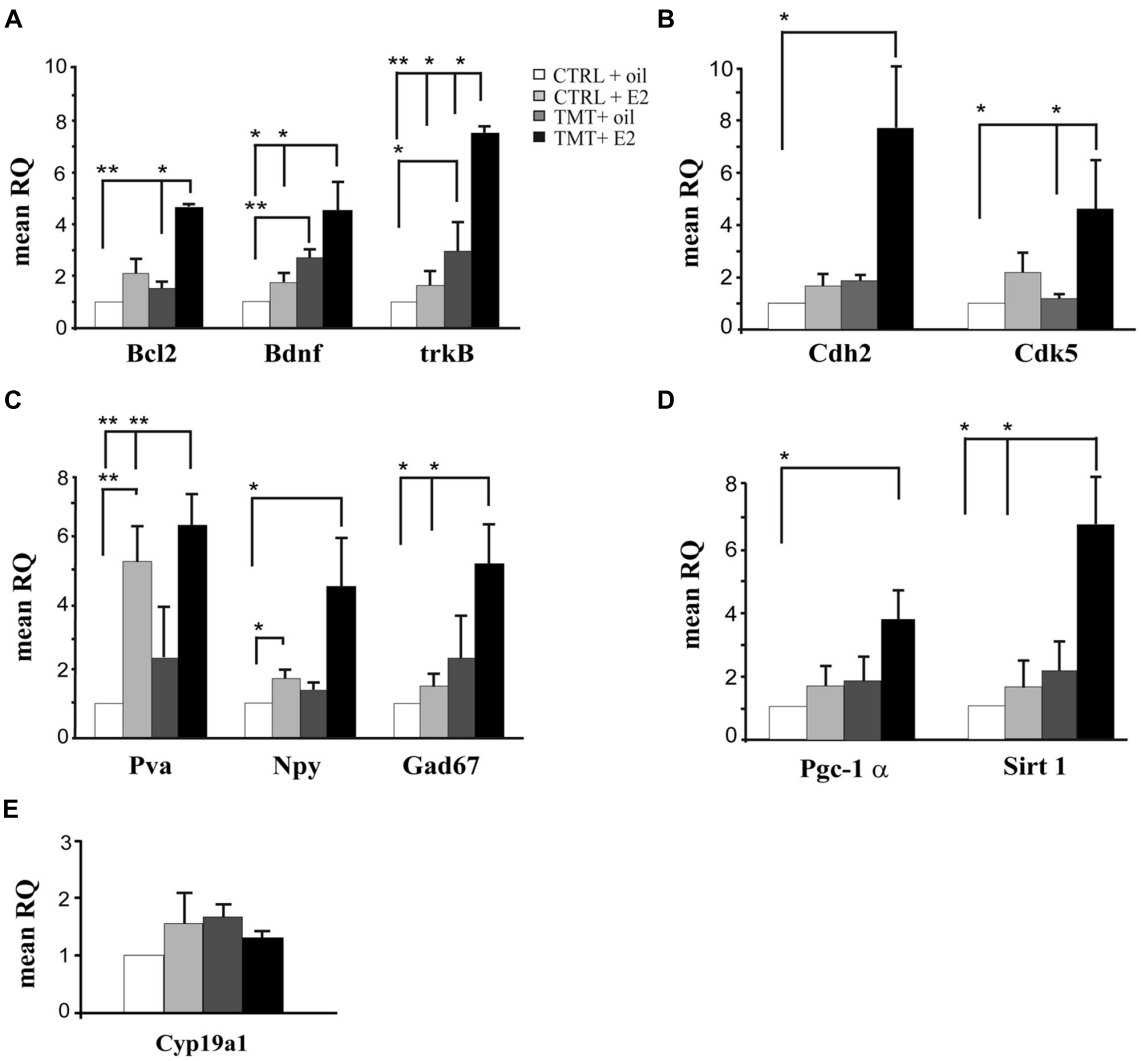
**Expression levels of genes modulated by E2 administration in the hippocampus of TMT-treated rats.** Bar graphs represent results of quantitative real time-PCR obtained using the DDCt method for the calculation of relative quantity (RQ) of the following genes: **(A)** Genes involved in neuroprotection (*Bcl2, Bdnf*, and *trkB*); **(B)** Genes involved in synaptogenesis (*Cdh2* and *Cdk5*); **(C)** Markers of interneurons (*Gad67, Pva, Npy*). **(D)** Genes involved in PV transcription (*Pgc-1α* and *Sirt 1*). **(E)** Gene related to local E2 biosynthesis*: aromatase (Cyp19a1)*. ^∗^*p* < 0.05, ^∗∗^*p* < 0.001, calculated on mean ΔCt across biological replicates.

In order to explore possible early neuroprotective events induced by E2 administration in TMT-treated rats, qPCR was used to amplify the following genes: the anti-apoptotic *Bcl2*, the neurotrophic factor *Bdnf* and the corresponding receptor *trkB*. A significant upregulation of *Bcl2* was detected in TMT + E2-treated rats compared with CTRL + oil- (*p* < 0.001) and with TMT + oil- (*p* < 0.05) treated groups (**Figure 1A**, Supplementary Table S2), while no significant difference could be detected in TMT + oil-treated animals when compared with both CTRL groups (*p* > 0.05). *Bdnf* appeared significantly increased in both groups of TMT-treated animals compared with the CTRL + oil-treated group (TMT + oil vs CTRL + oil *p* < 0.001; TMT + E2 vs CTRL + oil *p* < 0.05). The expression of *trkB* also appeared upregulated in both groups of TMT-treated animals compared with CTRL + oil-treated rats (*p* < 0.05), although its expression was significantly higher in the TMT + E2-treated animals than in all other groups (*p* < 0.05; **Figure 1A**, Supplementary Table S2).

Since the potential beneficial role of E2 may also be mediated by its effects on synaptic remodeling (Spencer et al., 2008; McEwen et al., 2012), the expression of two genes involved in synaptic plasticity, namely *Cdh2* (Tai et al., 2008; Bozdagi et al., 2010) and *Cdk5* (Lai and Ip, 2009) was also evaluated by qPCR. Results showed that E2 treatment induced a significant upregulation of *Cdk5* in TMT + E2 -treated rats compared with CTRL + oil- and TMT + oil-treated rats (*p* < 0.05); *Cdh2* was also significantly up-regulated in TMT + E2-treated rats compared with the CTRL + oil-treated group (*p* < 0.05; **Figure 1B**, Supplementary Table S2).

Expression of the interneuron markers GAD67, NPY and PV was also analyzed. qPCR analysis showed a significant increase in *Gad67* and *Pva* expression in TMT + E2-treated rats compared with both CTRL groups (*p* < 0.05 for *Gad67*; *p* < 0.001 for *Pva*); *Pva* was also up-regulated in the CTRL + E2-treated group compared with CTRL + oil-treated animals (*p* < 0.001), while no significant differences in *Gad67* and *Pva* gene expression were detectable between TMT + oil-treated animals and both CTRL groups or between TMT + E2- and TMT + oil-treated rats (*p* > 0.05). In addition, a significantly higher expression of the *Npy* gene was evident in TMT + E2- and in CTRL + E2-treated rats compared with the CTRL + oil group (*p* < 0.05; **Figure 1C**, Supplementary Table S2). Also in this case no significant difference in *Npy* gene expression was detectable between TMT + oil-treated animals and both control groups or between the TMT + E2- and the TMT + oil-treated group (*p* > 0.05).

The PGC-1α/Sirt 1 pathway, which is involved in PV transcription (Lucas et al., 2010), was upregulated by E2 treatment. In particular, the expression of *Pgc-1*α was significantly increased only in the TMT + E2-treated group compared with CTRL + oil-treated rats (*p* < 0.05); the *Sirt 1* gene expression was also significantly increased only in the TMT + E2-treated group compared with both control groups (*p* < 0.05). No significant difference in the *Pgc-1*α and *Sirt 1* gene expression was detectable between TMT + E2 and TMT + oil treated groups (*p* > 0.05; **Figure 1D**, Supplementary Table S2).

The possible modulation of hippocampal local E2 production was also explored through the analysis of the expression levels of *aromatase* (*Cyp19a1)*, the key enzyme involved in E2 biosynthesis, highly expressed in the rodent hippocampus (Hojo et al., 2004). qPCR analysis showed no significant differences in hippocampal *aromatase* expression levels among the four experimental groups (*p* > 0.05; **Figure 1E**, Supplementary Table S2).

### Persistent Effects Induced by E2 Administration

#### Effects of E2 Administration on TMT-induced Neuronal Death

To assess the characteristics of TMT-induced neurodegeneration and to evaluate the effects of E2 administration on TMT-induced neuronal death, we analyzed Nissl- and Fluoro Jade C-stained hippocampal sections from animals of the four experimental groups sacrificed 7 days after TMT or saline treatment, also performing unbiased quantitative analysis.

**FIGURE 2:**
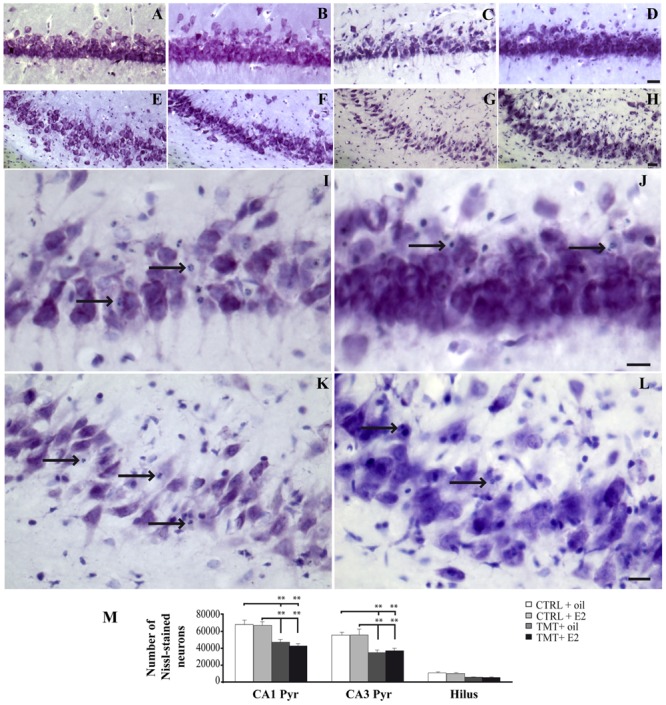
**Trimethyltin-induced hippocampal damage. (A–L)** Representative micrographs of Nissl-stained rat hippocampal sagittal sections from CA1 **(A–D,I,J)** and CA3 subfields **(E–H,K,L)** of CTRL + oil- **(A,E)**, CTRL + E2- **(B,F)**, TMT + oil- **(C,G,I,K)**, TMT + E2- **(D,H,J,L)** treated rats. Neuronal loss and apoptotic bodies are clearly detectable in CA3 and CA1 pyramidal neurons (arrows in **I–L**) of both TMT + oil- **(C,G,I,K)** and TMT + E2- **(D,H,J,L)** treated animals. Scale bar: 80 μm in **(A–H)**, 40 μm in **(I–L)**. **(M)** Bar graphs indicate quantitative analysis of Nissl-stained neurons in CA1 and CA3 pyramidal cell layer and hilus of the different experimental groups. A significant reduction in the number of Nissl-stained cells is evident in CA1 and CA3 pyramidal layers of both TMT-treated-groups compared with control groups. No differences are detectable between the two groups of TMT-treated rats. The values are given as means ± SE (^∗∗^*p* < 0.001).

Consistently with previous observations (Latini et al., 2010; Corvino et al., 2011, 2014), light microscope analysis of Nissl-stained samples showed mild to moderate neuronal death, selectively localized in the pyramidal layer of the CA1 (**Figures 2A–D,I,J**) and CA3 (**Figures 2E–H,K,L**) hippocampal subfields and in the hilus of both TMT-treated groups.

Unbiased stereological analysis followed by three-way RM ANOVA revealed a significant effect of TMT treatment (*F*_1,23_ = 50,3), hippocampal subfields (*F*_2,46_ = 320,5) and TMT^∗^hippocampal subfields interaction (*F*_2,46_ = 10,2). Tukey’s HSD *post hoc* test showed that a significantly lower number of surviving neurons was detectable in the CA1 and CA3 pyramidal layers of both TMT-treated groups compared with CTRL groups (*p* < 0.05). Despite the lower number of surviving neurons observed in the hilus of both TMT-treated groups, no significant differences were detectable compared with CTRL groups (*p* > 0.05; **Figure 2M**; Supplementary Table S3).

**FIGURE 3:**
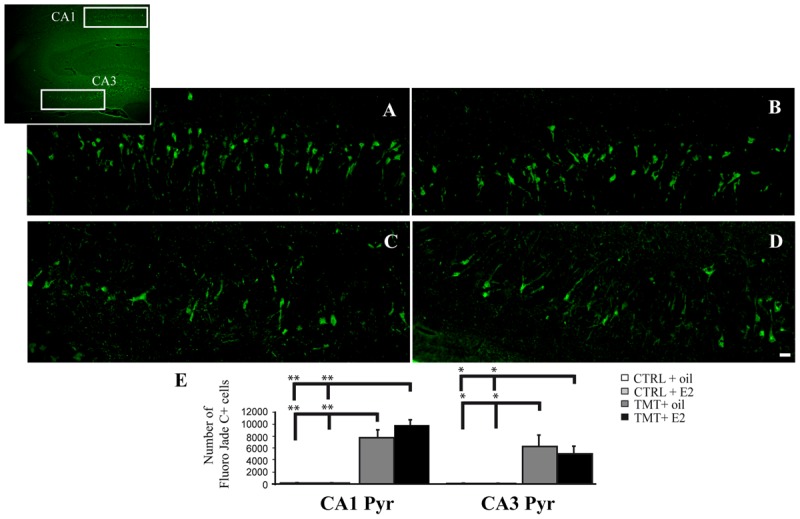
**Fluoro Jade C-stained degenerating neurons in the trimethyltin (TMT)-injured hippocampus.** Representative micrographs of Fluoro Jade C-stained rat hippocampal sagittal sections from CA1 **(A,B)** and CA3 subfields (**C,D**; as indicated in the box) of TMT + oil- **(A,C)**, TMT + E2- **(B,D)** treated rats. Degenerating neurons are evident in both TMT + oil- and TMT + E2-treated animals. Scale bar: 60 μm in **(A–D)**. **(E)** Bar graphs indicate quantitative analysis of Fluoro Jade C-stained neurons in CA1 and CA3 pyramidal cell layer of both TMT-treated groups. A significant difference in the number of Fluoro Jade C-stained cells is evident between TMT-treated rats and CTRL groups in both the CA1 and the CA3 regions, while no differences are detectable between TMT + oil- and TMT + E2-treated animals. The values are given as means ± SE (^∗^*p* < 0.05, ^∗∗^*p* < 0.001).

Fluorescent microscopy analysis of Fluoro Jade C-stained sections showed no stained neurons in the hippocampi of the two CTRL groups (not shown), as expected. Many stained degenerating neurons were evident in the CA1 (**Figures 3A,B**) and CA3 (**Figures 3C,D**) hippocampal regions of both TMT-treated groups, as expected (Corvino et al., 2012). Only a few scattered Fluoro Jade C-positive neurons were detectable in the hilus, without appreciable differences between TMT + E2- and TMT + oil-treated groups (not shown). In order to fulfill the requirements of the stereological approach, unbiased stereological analysis was performed only in the CA1 and CA3 subfields, which exhibited the higher density of stained cells. Three-way RM ANOVA revealed a significant effect of TMT treatment (*F*_1,31_ = 90,3). Tukey’s HSD *post hoc* test showed a significantly higher number of degenerating neurons in the CA1 and CA3 pyramidal layers of both TMT-treated groups compared both with CTRL + oil- (*p* < 0.001) and CTR + E2-treated group (*p* < 0.001); no differences were present between TMT + oil- and TMT + E2-treated animals in both CA1 and CA3 subfields (*p* > 0.05; **Figure 3E**; Supplementary Table S3).

#### Effects of E2 Administration on Hippocampal GAD67-Expression

**FIGURE 4:**
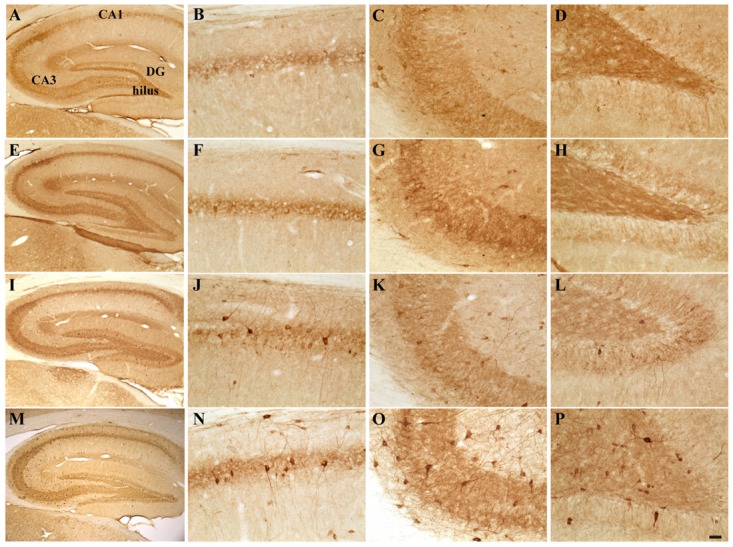
**Hippocampal distribution of glutamic acid decarboxylase 67 (GAD67)-immunoreactive interneurons in the different experimental groups.** Representative micrographs of GAD67-stained hippocampal sagittal sections from the whole hippocampus **(A,E,I,M)**, CA1 **(B,F,G,N)**, CA3 **(C,G,K,O)** and hilus **(D,H,L,P)** of CTRL + oil- **(A–D)**, CTRL + E2- **(E–H)**, TMT + oil- **(I–L)**, and TMT + E2- **(M–P)** treated rats. A higher number of GAD67-IR cells is evident in the CA1 pyramidal layer **(J,N)** and in the hilus **(L,P)** of both TMT-treated groups. GAD67 expression also appears markedly increased in the CA1 stratum oriens **(N)**, in the CA3 pyramidal layer **(O)**, in the DG and in the hilus **(P)** of TMT + E2-treated animals compared with all other groups. A darker staining of GAD67-IR neurons is evident in both groups of TMT-treated animals **(I,L,M,P)**. Scale bar: 200 μm in **(A,E,I,M)**; 50 μm in **(B–D,F–H,J–L,N–P)**.

Possible TMT- and/or E2-induced changes in the total number of GABAergic interneurons were explored through immunolabeling for the GABAergic marker GAD67. Light microscopy analysis of GAD67-stained hippocampal sections showed that, as described elsewhere (Hart et al., 2001), GAD67 immunoreactivity was detectable in all layers and subregions of the Cornu Ammonis, as well as in the hilus and in the granular layer of the DG; a GAD67-positive fiber plexus was also evident around the pyramidal cell somata located in the pyramidal layer of the whole Cornu Ammonis of animals from all experimental groups. A clear increase in the number of GAD67-IR cell bodies was appreciable in the hippocampi of both TMT-treated groups, more marked in the TMT + E2-treated group than in CTRL groups, associated with a darker immunostaining of positive cells (**Figure 4**).

**FIGURE 5:**
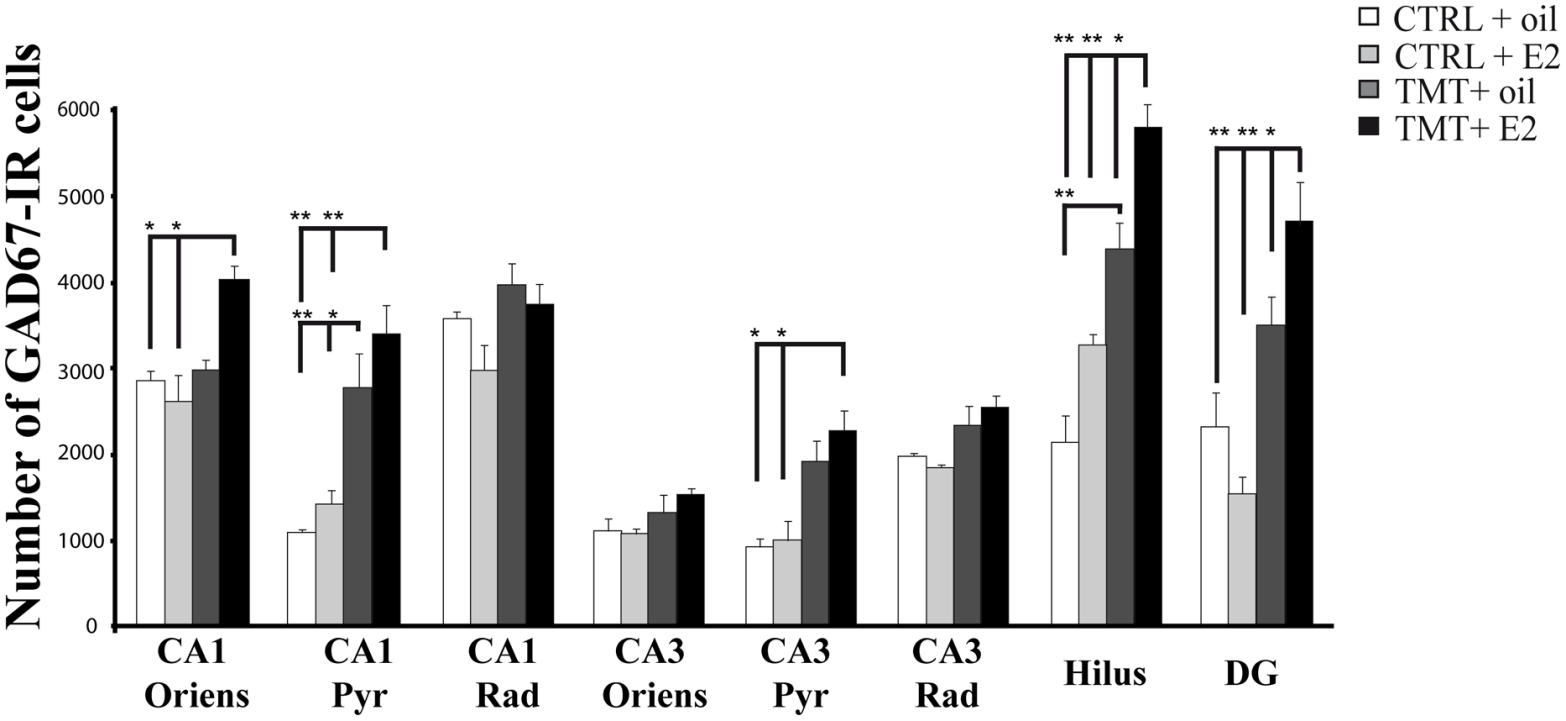
**Quantitative analysis of glutamic acid decarboxylase 67 (GAD67)-immunoreactive (IR) neurons in the hippocampus of animals of the different experimental groups.** Bar graphs indicate that a significantly higher number of GAD67-IR cells is present in the CA1 pyramidal layer and in the hilus of both TMT-treated groups. A further significant increase in the number of GAD67-IR neurons localized in the CA1 and CA3 pyramidal cell layers and in CA1 stratum oriens is evident in the TMT + E2-treated rats compared with both control groups and in the DG and hilus of the same group compared with all other groups. The values are given as means ± SE (^∗^*p* < 0.05, ^∗∗^*p* < 0.001).

Consistently, unbiased stereology followed by three-way RM ANOVA evidenced a significant effect of both TMT treatment (*F*_1,18_ = 81,6) and E2 administration (*F*_1,18_ = 4,5) on GAD67 expression. The effects of hippocampal subfields (*F*_7,126_ = 71,2), TMT^∗^E2 interaction (*F*_1,18_ = 5,8), TMT^∗^hippocampal subfields interaction (*F*_7,126_ = 13,1) and E2*hippocampal subfields interaction (*F*_7,126_ = 4,64) were also significant. Tukey’s HSD *post hoc* test evidenced the presence of a significantly higher number of GAD67-IR cells in the CA1 pyramidal layer of TMT + oil-treated animals compared with both control groups (*p* < 0.001 for TMT + oil-treated group vs CTRL + oil-treated rats, *p* < 0.05 for TMT + oil-treated group vs CTRL + E2-treated rats), and in the hilus of TMT + oil-treated animals compared with CTRL + oil-treated rats (*p* < 0.001; **Figure 5**, Supplementary Table S3).

E2 administration induced a further enhancement of GAD67 immunoreactivity in TMT-treated animals, with the TMT + E2-treated group showing a significantly higher number of GAD67-IR cells in the CA1 (*p* < 0.001) and CA3 (*p* < 0.05) pyramidal cell layers and in the CA1 stratum oriens (*p* < 0.05) compared with both control groups. In addition, the TMT + E2-treated animals also exhibited a higher number of GAD67-IR neurons in the DG and in the hilus than all other groups (*p* < 0.001 for TMT + E2-treated rats vs both control groups and *p* < 0.05 for TMT + E2-treated rats vs TMT + oil-treated rats; **Figure 5**, Supplementary Table S3).

#### Effects of E2 Administration on Hippocampal NPY and PV Expression

The distribution pattern of PV- and NPY-IR subpopulations was also evaluated in the light of data reporting that E2 administration could modulate their expression at the hippocampal level (Nakamura and McEwen, 2005; Rewal et al., 2005; Wu et al., 2014).

**FIGURE 6:**
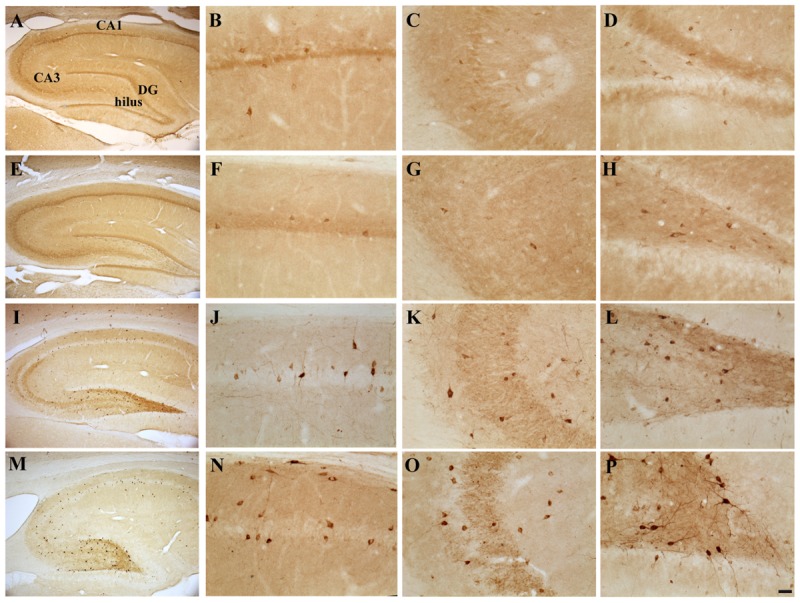
**Hippocampal distribution of neuropeptide Y (NPY)-immunoreactive interneurons in the different experimental groups.** Representative micrographs of NPY-stained hippocampal sagittal sections from the the whole hippocampus **(A,E,I,M)**, CA1 **(B,F,J,N)**, CA3 **(C,G,K,O)** and hilus **(D,H,L,P)** from CTRL + oil- **(A–D)**, CTRL + E2- **(E–H)**, TMT + oil- **(I–L)** and TMT + E2- **(M–P)**-treated rats. A higher number of darkly stained NPY-IR neurons is evident in CA1, CA3, and hilus of both TMT-treated groups compared with CTRL groups, being also more pronounced in the TMT + E2-treated animals. Scale bar: 200 μm in **(A,E,I,M)**; 50 μm in **(B–D,F–H,J–L,N–P)**.

Light microscopy analysis showed that, in both groups of CTRL animals, lightly stained NPY-positive neurons were detectable mainly in the CA1 stratum oriens and in the hilus; they were also present, albeit to a lesser extent, in the pyramidal layer of CA1, while only scattered NPY-IR cells were present in CA3 and in the DG, mainly localized in the subgranular zone, in line with previous reports (Deller and Leranth, 1990; Milner and Veznedaroglu, 1992). Both groups of TMT-treated animals exhibited a higher number of darkly stained NPY-IR neurons in the whole Cornu Ammonis and in the hilus, as expected (Ishida et al., 1997; Tsunashima et al., 1998; Ishikura et al., 2002); these findings were even more prominent in the TMT + E2-treated group (**Figure 6**).

Unbiased stereological cell counts were performed only in the hippocampal layers and subfields exhibiting a higher NPY-positive cell density, in order to fulfill the requirements of the stereological approach (namely the CA1 stratum oriens, pyramidal layer and hilus). Although in the CA3 subfield and in the DG NPY-IR cell density was higher in both TMT-treated groups than in controls (**Figures 6C,D,G,H,K,L,O,P**), unbiased stereology showed that the coefficient of error for estimations performed in these regions was >0.1 (Gundersen et al., 1999) and they were excluded.

**FIGURE 7:**
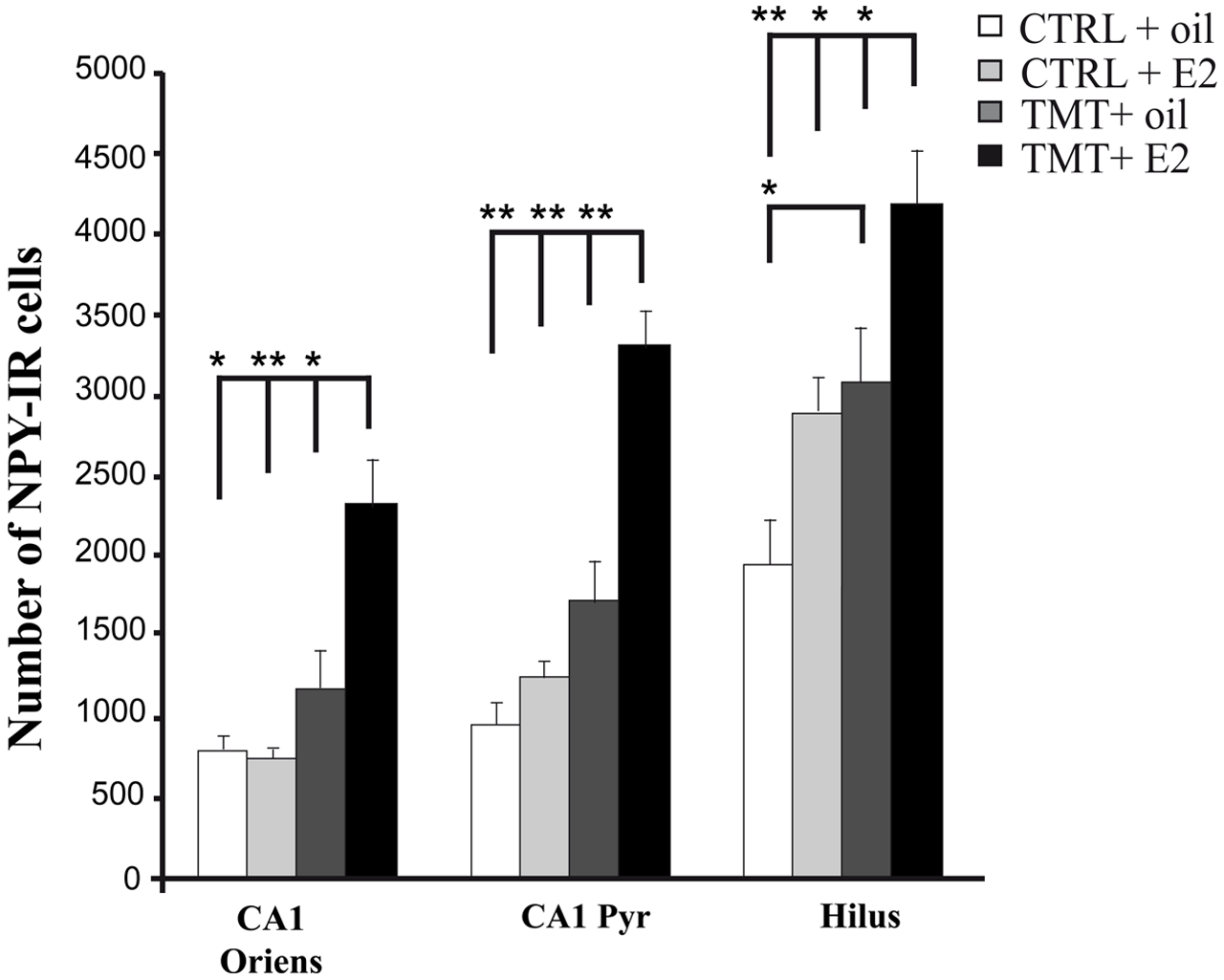
**Quantitative analysis of neuropeptide Y (NPY)-positive neurons in CA1 stratum oriens, CA1 pyramidal layer and hilus of the different experimental groups.** Bar graphs indicate that a significantly higher number of NPY-IR cells is evident in the hilus of the TMT + oil-treated group compared with CTRL + oil-treated animals. The TMT + E2-treated group shows a significant increase in the number of NPY-IR cells in CA1 stratum oriens, CA1 pyramidal layer and in the hilus compared with TMT + oil-treated animals and both control groups. The values are given as means ± SE (^∗^*p* < 0.05, ^∗∗^*p* < 0.001).

Three-way RM ANOVA statistical analysis evidenced a significant effect of both TMT administration (*F_1_*_,20_ = 45.16) and E2 treatment (*F*_1,20_ = 22.3) on NPY expression. The effects of hippocampal subfields (*F*_2,40_ = 66.3), as well as TMT^∗^E2 interaction (*F*_1,20_ = 6,28), were also present. Tukey’s HSD *post hoc* test showed a significant increase in the number of NPY-IR cells in the hilus of TMT + oil-treated animals compared with CTRL + oil-treated rats (*p* < 0.05) as expected (Ishida et al., 1997; Tsunashima et al., 1998; Ishikura et al., 2002) (**Figure 7**, Supplementary Table S3).

E2 administration resulted in a further enhancement of NPY expression in TMT-treated rats. Indeed, Tukey’s HSD *post hoc* test revealed a significant increase in the number of NPY-IR cells in the CA1 stratum oriens, the CA1 pyramidal layer and the hilus of TMT + E2-treated rats compared with TMT + oil-treated animals (*p* < 0.05 in the hilus and in CA1 stratum oriens; *p* < 0.001 in CA1 pyramidal layer), CTRL + oil-treated rats (*p* < 0.001 in the hilus and in CA1 pyramidal layer; *p* < 0.05 in CA1 stratum oriens), and CTRL + E2-treated animals (*p* < 0.001 in CA1 pyramidal layer and in CA1 stratum oriens; *p* < 0.05 in the hilus; **Figure 7**, Supplementary Table S3).

**FIGURE 8:**
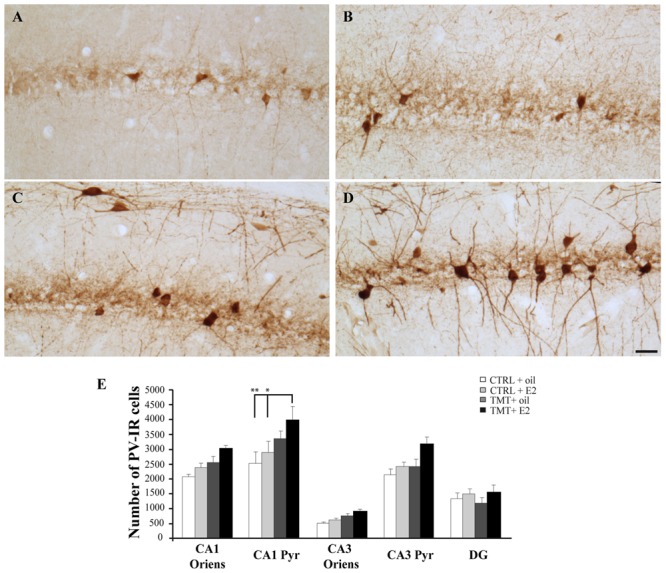
**Distribution and quantitative analysis of parvalbumin (PV)-immunoreactive (IR) neurons in the CA1 hippocampal region of the different experimental groups. (A–D)** Representative micrographs of PV-stained hippocampal sagittal sections from CA1 region of CTRL + oil- **(A)**, CTRL + E2- **(B)**, TMT + oil- **(C)**, and TMT + E2- **(D)** treated rats. A higher number of PV-IR cells is detectable in the CA1 pyramidal layer of TMT + E2-treated rats compared with both control groups. Scale bar: 50 μm. **(E)** Number of PV-IR neurons in different hippocampal subfields of the different experimental groups. A significantly higher number of PV-IR cells is evident in the CA1 pyramidal layer of TMT + E2-treated rats compared with both control groups. The values are given as means ± S.E. (^∗^*p* < 0.05, ^∗∗^*p* < 0.001).

Light microscope analysis of PV immunoreactivity in both TMT-treated and CTRL groups revealed the presence of PV-IR cell bodies located in all hippocampal layers, mainly localized in the stratum oriens and pyramidal cell layers of the Cornu Ammonis and in the granule cell layer of the DG, as well as the presence of a PV-IR fiber plexus in the pyramidal layer, reflecting the cell distribution described elsewhere (Andressen et al., 1993; Geloso et al., 1996, 1998). No differences in the number or distribution pattern of PV-IR cells were detectable by light microscopy examination between TMT + oil-treated animals and both CTRL groups, as expected (Geloso et al., 1996), while an increased number of PV-IR neurons was detectable in the CA1 pyramidal cell layer of TMT + E2-treated animals compared with all other groups (**Figures 8A–D**). In particular, stereological cell counts followed by three-way RM ANOVA statistical analysis indicated significant factors: TMT (*F*_1,29_ = 8,4), E2 (*F*_1,29_ = 5,5), hippocampal subfields (*F*_4,116_ = 92,5), TMT^∗^hippocampal subfields (*F*_4,116_ = 3,5). Tukey’s HSD *post hoc* test evidenced a significant increase in the number of PV-IR cells in the CA1 pyramidal layer of TMT + E2-treated rats compared with both control groups (*p* < 0.001 for TMT + E2-treated rats vs CTRL + oil-treated group; *p* < 0.05 for TMT + E2-treated rats vs CTRL + E2-treated group; **Figure 8E**, Supplementary Table S3).

**FIGURE 9:**
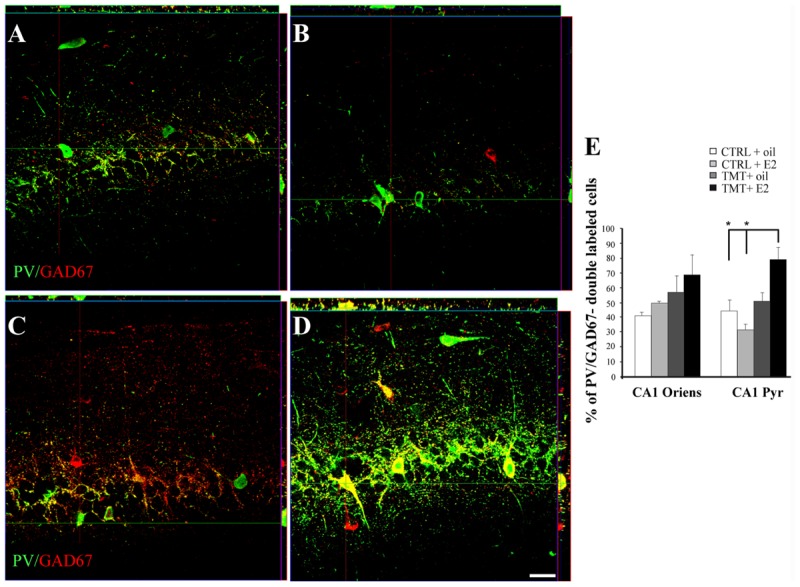
**Co-expression of the interneuronal markers parvalbumin (PV) and glutamic acid decarboxylase (GAD) 67 in the CA1 hippocampal region of the different experimental groups.** Representative confocal reconstructed orthogonal images, as viewed in the *x-z* (top) and *y-z* (right) planes, of hippocampal sagittal sections from the CA1 subfield of CTRL + oil- **(A)**, CTRL + E2- **(B)**, TMT + oil- **(C)**, TMT + E2- **(D)** treated rats double-labeled for GAD67 (red) and PV (green). The colocalization of the two markers is considerably more evident in TMT + E2 treated animals. Scale bar: 40 μm. **(E)** Bar graphs indicating the percentage of PV/GAD67-double-stained cells in CA1 oriens and pyramidal layers. The percentage of PV/GAD67-double-stained cells in CA1 pyramidal layer is higher in TMT + E2-treated rats compared with both control groups. The values are given as means ± SE (^∗^*p* < 0.05).

Confocal microscope quantitative analysis of NPY/GAD67 and PV/GAD67 double-labeled cells was performed in the specific hippocampal subfields exhibiting significant changes in the expression of NPY or PV immunoreactivity, to analyze possible differences in GAD67 expression in these interneuron subpopulations among the four experimental groups. In particular, quantitative analysis of PV/GAD67 double-labeled cells performed in CA1 (stratum oriens and pyramidal layer) revealed a significantly higher percentage of PV/GAD67 double-labeled cells in the CA1 pyramidal layer of TMT + E2-treated rats compared with both control groups (Two-way ANOVA *F*_1,14_ = 14,1 for TMT vs CTRL and *F*_1,14_ = 7,9 for interaction; Tukey’s HSD *post hoc* test *p* < 0.05; **Figures 9A–D**). No significant differences in the percentage of PV/GAD67 double-labeled cells were evident in the CA1 stratum oriens (Two-way ANOVA *p* > 0.05; **Figure 9E**).

Quantitative analysis of NPY/GAD67 double-labeled cells performed in the CA1 stratum oriens, CA1 pyramidal layer and in the hilus showed no significant differences among groups (Two-way ANOVA *p* > 0.05; Supplementary Figure S1).

## Discussion

Modulation of the GABAergic system is a remarkable aspect of neuroprotective strategies, including those based on E2 administration (Iuvone et al., 1996; Dell’Anna et al., 1997; Hart et al., 2001; Czeh et al., 2005; Velísková and Velísek, 2007; Ledoux et al., 2009; Ohira et al., 2013), due to the major role exerted by interneurons in maintaining the appropriate excitatory/inhibitory synaptic balance, which is critical for hippocampal information processing (Buzsáki and Chrobak, 1995; Evstratova and Tóth, 2014).

In this regard, the present study shows that E2 administration during hippocampal neurodegeneration induced by the neurotoxicant TMT causes the early activation of genes involved in neuroprotection and synaptogenesis, as well as persistent regionally restricted changes in GAD67-IR interneuron subpopulation size, which also involve NPY- and PV-expressing cells. In line with the notion that one of the mechanisms through which estrogens exert neuroprotection involves the control of cell death (Amantea et al., 2005; Arevalo et al., 2015), we observed that E2 administration causes an early and significant upregulation of the anti-apoptotic gene *Bcl2* (Sastry and Rao, 2000) in the hippocampi of TMT-treated animals. The BDNF pathway, which is crucially involved in neuroprotection in different pathological conditions (Reibel et al., 2001; Almeida et al., 2005), including seizures (Binder et al., 2001), appears to be modulated following TMT-treatment, in line with previous observations (Andersson et al., 1997), possibly as a result of the activation of endogenous protective mechanisms. Interestingly, E2 administration induced, in TMT-treated rats, significantly higher levels of *trkB*. This is consistent with evidence indicating that the BDNF pathway is one of the molecular mediators of E2-induced effects in the hippocampus. However, although it has been considered principally involved in E2-mediated neuroprotective and homeostatic functions (Arevalo et al., 2015), a dual role of this neurotrophin has also been proposed. Some findings suggest that BDNF may also increase neuronal excitability, even contributing to epileptogenesis (Binder et al., 2001), and a possible detrimental effect cannot be excluded.

Although our findings suggest that E2 administration results in the early activation of molecular pathways possibly aimed at preventing TMT-induced neuronal damage, we found no significant differences in the extent of neuronal death between the two groups of TMT-treated animals, as evidenced by unbiased stereology on both Nissl and Fluoro Jade C staining performed at the later time point. Previous observations by other groups indicate that scheduled treatment based on high doses of E2, as in the present study, are effective in reducing neuronal death in different experimental conditions, including ischemia and kainic acid-induced seizures (Azcoitia et al., 1998; Picazo et al., 2003; Inagaki and Etgen, 2013). Although the reason for this discrepancy remains uncertain, differences in the pathogenic events that characterize the different experimental models may account for the ineffectiveness of E2-induced neuroprotective pathways on neuronal survival in TMT intoxication. Since specific features of the microenvironment, including changes in neural activity, can reverse the neuroprotective properties of some neurotrophic agents, including also BDNF (Guo et al., 2014), we may speculate that the persistence of cellular stress (Fulda et al., 2010) could explain our results.

The BDNF pathway is also believed to play a relevant role in E2-induced synaptogenesis (Spencer et al., 2008) at hippocampal level (Scharfman and MacLusky, 2006; Spencer et al., 2008).

In this regard, TMT + E2-treated animals also show an upregulation of other relevant players in the molecular cascade leading to morphological plasticity, namely *Cdk5*, which plays a role in the regulation of dendritic spine formation (Fischer et al., 2003; Lai and Ip, 2009), and the *Cdh2* gene, a synaptic adhesion molecule involved in the formation and maintenance of synaptic structure and function (Tai et al., 2008; Bozdagi et al., 2010; Mendez et al., 2010). Both molecules are also known to interact with the BDNF signaling cascade. Indeed, Cdk5-mediated phosphorylation of TrkB has been proposed to be essential in BDNF-induced dendritic growth (Cheung et al., 2007) and the involvement of Cdh2 in the molecular pathways activated during the BDNF/TrkB-induced effects on synaptogenesis has also been described (Bamji et al., 2006).

E2-mediated plastic phenomena, may be also regulated by locally synthetized estrogens (Hojo et al., 2004, 2008; Rune and Frotcher, 2005; for review, see Fester and Rune, 2015), which, at the hippocampal level, are produced by neurons and, under pathological conditions, also by reactive astroglial cells (Naftolin et al., 1996; Garcia-Segura et al., 1999; Wehrenberg et al., 2001; Prange-Kiel et al., 2003). Since possible interactions between the pathways of locally produced and exogenous E2 have been suggested (Iivonen et al., 2006; Pietranera et al., 2008), basal levels of local E2 production were evaluated, through the analysis of the expression levels of *aromatase*, key player in hippocampal E2 production (Hojo et al., 2004). Our results indicate that *aromatase* expression is unaffected by the neurotoxicant administration in the early phases of TMT-induced hippocampal injury, as well as by the concomitant E2 treatment. Since basal levels of endogenous E2 have been suggested to exert a relevant role on exogenous E2-mediated effects (Chamniansawat and Chongthammakun, 2012), an interaction between the two pathways cannot be excluded.

A large body of evidence shows that, through genomic and non-genomic mechanisms (Wong and Moss, 1992; Foy et al., 1999; Liu et al., 2012), one of the main effects of E2 on the hippocampus is the modulation of neuronal excitability, exerted through the activation of NMDA receptors, especially in the CA1 subfield (Woolley et al., 1997; McEwen et al., 2012), which is also believed to underlie synaptogenesis (Woolley et al., 1997; McEwen et al., 2012).

Enhanced E2-induced excitability is accompanied by increased GABAergic transmission (Murphy et al., 1998), which results in the modulation of GAD67 expression (Weiland, 1992; Nakamura et al., 2004; Spencer et al., 2008). Moreover, the presence of estrogen receptors in the hippocampal GABAergic interneurons (McEwen and Alves, 1999; Hart et al., 2001; Blurton-Jones and Tuszynski, 2002; Waters et al., 2015) enables them to play a pivotal role in estrogen-mediated plastic events in the adult hippocampus (Spencer et al., 2008).

Although TMT does not appear to be excitatory in nature (Allen and Fonnum, 1984; Koczyk, 1996), it has been suggested that excitotoxicity may be involved in TMT-induced neuronal death (Koczyk, 1996; Nishimura et al., 2001; Geloso et al., 2011). The involvement of the GABAergic system has also been reported (Wilson et al., 1986; Nishimura et al., 2001). Our results indicate that TMT + oil-treated animals exhibit a marked increase in GAD67 expression in selected hippocampal subfields (namely the CA1 pyramidal cell layer and the hilus), in line with previous reports (Nishimura et al., 2001). Interestingly, E2 administration induced, in the TMT-injured hippocampus, not only an early upregulation of the *Gad67* gene, but also a further increase in GAD67 immunoreactivity, likely suggesting an E2-mediated increase in GAD67 levels. GAD67 levels have been directly related to the efficacy of synaptic transmission in GABAergic interneurons (Lazarus et al., 2015). Increased GAD67 expression may thus reflect a general cellular response to injury and/or it may represent an attempt to increase the production and, possibly, the release of GABA (Czeh et al., 2005). The increased GAD67 immunoreactivity appears as a persistent and regionally specific effect. In particular, TMT + E2-treated animals show a significantly higher number of GAD67-IR cells in the CA1 stratum oriens, where dendrite-projecting interneurons are located, which are known to control the input of principal cells and the propagation of calcium currents from the dendrite to the soma (Sik et al., 1995; Cossart et al., 2001), and in the CA3 pyramidal layer, one of the principal sites where the neurotoxicant exerts its earliest and most severe effects (Koczyk, 1996; Geloso et al., 2011) and where GABAergic basket cells are located (Sik et al., 1995). In the same group, the enhancement of GAD67 expression was also detectable in the hilus, in which both interneurons (Mott et al., 1997) and mossy cells (Jinde et al., 2013) are involved in the control of dentate granule cell excitability, and in the DG, commonly considered a gate structure controlling incoming inputs to the hippocampus (Hsu, 2007).

In line with previous evidence (Velísková and Velísek, 2007; Velisek et al., 2013), our data indicate that E2 administration also increases *Npy* gene expression levels in both CTRL + E2- and TMT + E2-treated groups, which results, in TMT-treated animals, in a further and more extensive enhancement of NPY immunoreactivity. It is known that the NPY system undergoes profound changes during many neurodegenerative diseases, as well as in experimental models of temporal lobe epilepsy (Decressac and Barker, 2012; Malva et al., 2012), including the TMT-induced model of hippocampal injury (Ishida et al., 1997; Tsunashima et al., 1998; Ishikura et al., 2002). This feature has also been confirmed by the present study. In this regard, E2 administration results in a further enhancement of NPY expression in TMT-treated rats, with a regional distribution that parallels the increased expression of GAD67 observed in the same group, involving, in addition, also the CA1 pyramidal layer, the particular site of estrogen activity (Nakamura and McEwen, 2005; Brann et al., 2007). We may hypothesize that the two findings are part of the same phenomenon, as also indicated by the lack of significant differences in the percentage of NPY/GAD67 double-labeled cells detected in the same regions among the different experimental groups.

Together with an impairment in neurotransmission, the occurrence of seizures has also been described in TMT-treated rats, mainly in the time frame between 4 and 16 days after administration of the neurotoxicant (for review, see Koczyk, 1996; Geloso et al., 2011; Corvino et al., 2013; Lattanzi et al., 2013). Therefore, taking into account the suggestion that estrogens exert their neuroprotective effects by enhancing, possibly through BDNF induction, the expression of NPY, whose anticonvulsant and antiapoptotic properties are widely recognized (Wu and Li, 2005; Smialowska et al., 2009; Corvino et al., 2012), a possible homeostatic and reparative role of this phenomenon may be speculated.

Our findings also point to the involvement of the PV-expressing subpopulation in the changes induced by E2 administration in the TMT-injured hippocampus. E2 administration induces the early and significant upregulation of the *Pva* gene both in CTRL and in TMT-treated animals. In the latter group, interestingly, this finding is further supported by the upregulation of molecular pathways involved in PV expression, such as *Pgc-1*α, required for both mRNA and protein expression of PV in the hippocampus (Lucas et al., 2010; Jinde et al., 2013) and a master regulator of mitochondrial biogenesis (St-Pierre et al., 2006), and *Sirt 1*, a histone deacetylase that directly regulates the activity of PGC-1α (Rodgers et al., 2008; Aquilano et al., 2010). This is not surprising, since PV-expressing interneurons are known to express estrogen receptors (Blurton-Jones and Tuszynski, 2002; Higaki et al., 2012) and changes in size of the PV-expressing subpopulation following E2 treatment have previously been reported in different brain regions (Rewal et al., 2005; Macrì et al., 2010; Sotonyi et al., 2010; Koh, 2014), as well as in non-neural tissues (Wirakiat et al., 2012).

In this regard, a putative link between this effect and the observed modulation of the BDNF/TrkB pathway cannot be excluded, due to the described influence exerted by the neurotrophin on the transcription of proteins involved in GABAergic transmission, including PV and NPY (Glorioso et al., 2006).

Upregulation of the *Pva gene* and related pathways results, in TMT-treated animals, in increased PV immunoreactivity, which, also in this case, selectively involves the CA1 pyramidal cell layer. Changes in size of the PV-expressing subpopulation may reflect variations in the cellular content of this calcium-binding protein (Scotti et al., 1997), which in turn have been related to variations in the activity state of these interneurons (Scotti et al., 1997; Vreugdenhilm et al., 2003; Donato et al., 2013; Urakawa et al., 2013). Interestingly, a higher percentage of PV-IR cells co-expressing the GABAergic marker GAD67 is detectable in the CA1 pyramidal cell layer of the same experimental group.

GAD67 levels are thought to reflect cellular and vesicular GABA contents, as well as changes in the activity state of GABAergic interneurons (Esclapez and Houser, 1999; Ramirez and Gutierrez, 2001; Lazarus et al., 2015). GAD67 also regulates axon branching and perisomatic bouton formation in PV-expressing basket neurons (Chattopadhyaya et al., 2007), contributing to the functional state and plasticity in these cells (Lazarus et al., 2015). Our findings therefore suggest the occurrence of molecular events possibly related to functional changes in PV-positive hippocampal cells.

Taken together, our results indicate that although E2 administration fails to counteract TMT-induced neuronal death, it mediates the expression of molecules related to neuronal plasticity and to inhibitory neurotransmission, and that this is associated with persistent phenotypic changes in the size of different GABAergic subpopulations. Among the latter, NPY- and PV-IR neurons, in particular, can be selectively affected in many pathologic conditions, including Angelman syndrome (Godavarthi et al., 2014), Alzheimer’s disease (Ramos et al., 2006), schizophrenia (Stansfield et al., 2015), aging (Pugliese et al., 2004; Kuruba et al., 2011), and seizures (Sun et al., 2007). Neuroprotective strategies that lead eventually to a modulation of the neurochemical features of these interneurons may be potentially relevant for new therapeutic approaches in brain disease.

## Author Contributions

MCG made substantial contributions to both the conception and design of the work; she contributed to the acquisition, analysis, and interpretation of data. She drafted the work and revised it critically. She approved the final version to be published. She agrees to be accountable for all aspects of the work and for ensuring that questions related to the accuracy or integrity of any part of the work are appropriately investigated and resolved.

VC gave substantial contributions to the conception and design of the experiments; she contributed to the acquisition, analysis, and interpretation of data. She drafted the work and revised it critically. She approved the final version to be published. She agrees to be accountable for all aspects of the work and for ensuring that questions related to the accuracy or integrity of any part of the work are appropriately investigated and resolved.

VDM performed the experiments and made substantial contributions to the acquisition of data. She drafted the work. She approved the final version to be published. She agrees to be accountable for all aspects of the work and for ensuring that questions related to the accuracy or integrity of any part of the work are appropriately investigated and resolved.

EM performed the experiments and made substantial contributions to the acquisition of data. She drafted the work. She approved the final version to be published. She agrees to be accountable for all aspects of the work and for ensuring that questions related to the accuracy or integrity of any part of the work are appropriately investigated and resolved.

FB made substantial contributions to the interpretation of data; he critically revised the manuscript. He approved the final version to be published. He agrees to be accountable for all aspects of the work and for ensuring that questions related to the accuracy or integrity of any part of the work are appropriately investigated and resolved.

WL made substantial contributions to the analysis of data; she critically revised the manuscript. She approved the final version to be published. She agrees to be accountable for all aspects of the work and for ensuring that questions related to the accuracy or integrity of any part of the work are appropriately investigated and resolved.

FM provided substantial contributions to the design of the work; he contributed to the interpretation of data. He critically revised the work He approved the final version to be published. He agrees to be accountable for all aspects of the work and for ensuring that questions related to the accuracy or integrity of any part of the work are appropriately investigated and resolved.

## Conflict of Interest Statement

The authors declare that the research was conducted in the absence of any commercial or financial relationships that could be construed as a potential conflict of interest.

## Abbreviations

CTRL: control
DG: Dentate gyrus
E2: 17β-estradiol
i.p.: intra-peritoneal
IR: immunoreactive
PV: parvalbumin
TMT: trimethyltin
