# Supplementary material for: Estrogen administration modulates hippocampal GABAergic subpopulations in the hippocampus of trimethyltin-treated rats
Source: Front Cell Neurosci. 2015 Nov 5;9:433. doi: 10.3389/fncel.2015.00433 (PMC4633568; doi:10.3389/fncel.2015.00433)
Supplement: Supplementary file 1 [file Data_Sheet_1.PDF]

Estrogen administration modulates hippocampal GABAergic subpopulations in the hippocampus of trimethyltin-treated rats

Valentina Corvino[1¶], Valentina Di Maria[1¶], Elisa Marchese[1¶], Wanda Lattanzi[1], Filippo Biamonte[2], Fabrizio Michetti[1&], Maria Concetta Geloso*[1&]

* Correspondence:
Dr. Maria Concetta Geloso
Università Cattolica del Sacro Cuore
Institute of Anatomy and Cell Biology
Largo F. Vito, n° 1
00168, Rome, Italy
mc.geloso@rm.unicatt.it

Supplementary Table 1. Oligonucleotide primer sequences.

Gene Symbol	Forward Primer	Reverse Primer
**Bcl-2**	5’-tcaaagaaggccacaatcct-3’	5′-ggtggtggaggaactcttca-3’
**Bdnf**	5’-cgaaccttctggtcctcatc-3’	5’-tggctgacacttttgagcac-3’
**Ntrk2**	5’-caagctgacgagtttgtcca-3’	5’-ttacccgtcaggatcaggtc-3’
**Cdh2**	5’-acgggcagatcaccactatc-3’	5’-tgacatttggcgactctctg-3’
**Cdk5**	5’-gcaatgatgtggatgaccag -3’	5’-ggtgtccctagcagtcgaaa-3’
**Pva**	5’-cgaccacaaaaagttcttcca-3’	5’-ccgcactctttttctcagg-3’
**Gad1**	5’-agtgttctgccatcctggtc-3’	5’-atctggttgcatccttggag-3’
**Npy**	5’-tactccgctctgcgacacta-3’	5’-tctcagggctggatctcttg-3’
**Ppargc1a**	5’-aaggtccccaggcagtagat-3’	5’-cggtgtctgtagtggcttga-3’
**Sirt1**	5’-atgacagagcatcacacgca-3’	5’-tatggacctatccgtggcct-3’
**Cyp19a1**	5’- ctcctcctgattcggaattgt-3’	5’-tctgccatgggaaatgagag-3’
**Gapdh**	5’-ctgaggaccaggttgtctcc-3’	5’-ggaagaatgggagttgctgt-3’

Supplementary Table 2. Values of mean ΔCt, SD, SEM and p referred to qPCR results.

Sample	Pva			
	MEAN ΔCt	SD	SEM	p-value
CTRL+oil	8.973153114	0.30542	0.15271	TMT+oil vs CTRL+oil: 0.3274
CTRL+E2	6.729292826	0.66774	0.33387	TMT+E2 vs CTRL+oil:0.0001
TMT+oil	8.190736413	1.43553	0.71777	CTRL+E2 vs CTRL+oil: 0.0009
TMT+E2	6.401052475	0.41331	0.20666	TMT+E2 vs TMT+oil:0.0536
				TMT+E2 vs CTRL+E2:0.0001

Sample	Npy			
	MEAN ΔCt	SD	SEM	p-value
CTRL+oil	7.966562748	0.25261	0.12630	TMT+oil vs CTRL+oil:0.1696
CTRL+E2	7.241355777	0.45444	0.22722	TMT+E2 vs CTRL+oil:0.0339
TMT+oil	7.5628366849	0.45104	0.22552	CTRL+E2 vs CTRL+oil:0.0316
TMT+E2	5.716437459	1.62472	0.81236	TMT+E2 vs TMT+oil:0.071
				TMT+E2 vs CTRL+E2:0.1206

Sample	Gad67			
	MEAN ΔCt	SD	SEM	p-value
CTRL+oil	7.628402948	0.53189	0.26594	TMT+oil vs CTRL+oil:0.2433
CTRL+E2	7.209826827	0.79588	0.39794	TMT+E2 vs CTRL+oil:0.0041
TMT+oil	6.860171199	1.29985	0.64992	CTRL+E2 vs CTRL+oil:0.4155
TMT+E2	5.442192285	0.81002	0.40501	TMT+E2 vs TMT+oil:0.1136
				TMT+E2 vs CTRL+E2:0.028

Sample	Cdh2			
	MEAN ΔCt	SD	SEM	p-value
CTRL+oil	8.272434473	0.52935	0.264667	TMT+oil vs CTRL+oil:0.1284
CTRL+E2	7.528994918	0.77108	0.38554	TMT+E2 vs CTRL+oil:0.0238
TMT+oil	6.941956639	1.41367	0.70683	CTRL+E2 vs CTRL+oil:0.1653
TMT+E2	5.40842402	1.82985	0.91493	TMT+E2 vs TMT+oil:0.2331
				TMT+E2 vs CTRL+E2:0.0766

Sample	Pgc1α			
	MEAN ΔCt	SD	SEM	p-value
CTRL+oil	9.440998077	0.65687	0.32844	TMT+oil vs CTRL+oil:0.8280
CTRL+E2	8.46717608	1.64128	0.82064	TMT+E2 vs CTRL+oil:0.031
TMT+oil	8.772238893	0.90439	0.45220	CTRL+E2 vs CTRL+oil:0.3130
TMT+E2	7.272240043	1.39029	0.69515	TMT+E2 vs TMT+oil:0.1192
				TMT+E2 vs CTRL+E2:0.3091

Sample	Sirt1			
	MEAN ΔCt	SD	SEM	p-value
CTRL+oil	6.856110121	0.63784	0.31892	TMT+oil vs CTRL+oil:0.71105
CTRL+E2	6.632305026	1.71053	0.85527	TMT+E2 vs CTRL+oil:0.006
TMT+oil	6.414668432	2.17692	1.08846	CTRL+E2 vs CTRL+oil:0.8141
TMT+E2	4.060698357	1.17434	0.58717	TMT+E2 vs TMT+oil:0.1057
				TMT+E2 vs CTRL+E2:0.047

Sample	Cdk5			
	MEAN ΔCt	SD	SEM	p-value
CTRL+oil	9.150751829	0.15861	0.07930	TMT+oil vs CTRL+oil: 0.1573
CTRL+E2	8.231392026	1.05647	0.52824	TMT+E2 vs CTRL+oil:0.0212
TMT+oil	8.919179639	1.19035	0.59518	CTRL+E2 vs CTRL+oil: 0.1383
TMT+E2	7.072318196	1.30587	0.65293	TMT+E2 vs TMT+oil:0.031
				TMT+E2 vs CTRL+E2:0.2167

Sample	Bdnf			
	MEAN ΔCt	SD	SEM	p-value
CTRL+oil	7.981389046	0.05389	0.02695	TMT+oil vs CTRL+oil:0.0001
CTRL+E2	7.255723405	0.62433	0.31216	TMT+E2 vs CTRL+oil:0.0048
TMT+oil	6.500759713	0.34069	0.17034	CTRL+E2 vs CTRL+oil:0.06
TMT+E2	5.603528621	1.09290	0.54645	TMT+E2 vs TMT+oil:0.168
				TMT+E2 vs CTRL+E2:0.04

Sample	trkB

	MEAN ΔCt	SD	SEM	p-value
CTRL+oil	5.280315284	0.17041	0.0854	TMT+oil vs CTRL+oil:0.036
CTRL+E2	4.971614101	1.30278	0.65139	TMT+E2 vs CTRL+oil:0.0001
TMT+oil	3.961476509	0.96927	0.48464	CTRL+E2 vs CTRL+oil:0.654
TMT+E2	2.35406139	0.10589	0.05294	TMT+E2 vs TMT+oil:0.016
				TMT+E2 vs CTRL+E2:0.007

Sample	Bcl2			
	MEAN ΔCt	SD	SEM	p-value
CTRL+oil	12.57779892	0.42319	0.21160	TMT+oil vs CTRL+oil:0.1472
CTRL+E2	11.35615399	1.36523	0.68262	TMT+E2 vs CTRL+oil:0.0001
TMT+oil	11.66001823	1.01843	0.50922	CTRL+E2 vs CTRL+oil:0.1383
TMT+E2	10.34145454	0.05696	0.02848	TMT+E2 vs TMT+oil:0.041
				TMT+E2 vs CTRL+E2:0.1880

Sample	Cyp19a1			
	MEAN ΔCt	SD	SEM	p-value
CTRL+oil	14.50050	0.49845	0.24922	TMT+oil vs CTRL+oil:0.07
CTRL+E2	14.06775	0.87820	0.43910	TMT+E2 vs CTRL+oil:0.2319
TMT+oil	13.78250	0.40441	0.20221	CTRL+E2 vs CTRL+oil:0.4243
TMT+E2	14.13375	0.23630	0.11813	TMT+E2 vs TMT+oil:0.1843
				TMT+E2 vs CTRL+E2:0.8893

Supplementary Table 3. Stereological cell count results

	CTRL+oil	CTRL+E2	TMT+oil	TMT+E2
**NISSL-Stained neurons**				
**CA1 pyramidal layer**	67359,6±5177	62149,3±3680,3	46458,6±3447	42779,2±2428
**CA3 pyramidal layer**	54847,7±3562,8	56862,7±551,6	34338,9±3243,6	35768,2±2554,3
**Hilus**	16045±893,9	13372,3±491	8461,6±557,6	8188,1±687,6
**FLUORO JADE C- stained neurons**				
**CA1 pyramidal layer**	0±0	0±0	7735,5±1747,5	9810,9±929,8
**CA3 pyramidal layer**	0±0	0±0	6250,7±1207,15	4907,29±1995,9
**GAD 67-IR cells**				
**CA1 stratum oriens**	2841,02±135	2612,07± 315	2979,38± 127	4030,47± 165
**CA1 pyramidal layer**	1099,22±30	1404,37±180	2766,1±400	3398,8±342
**CA1 stratum radiatum**	3576,6±84	2959,7±317	3957,2±269	3748,8±230
**CA3 stratum oriens**	1118,4±143	1079,5±62	1332,2±212	1542,2±60
**CA3 pyramidal layer**	926,9±106	994,2±247	1912,9±249	2277,7±239
**Ca3 stratum radiatum**	1976,9±46	1834,2±61	2329,7±242	2548,4±137
**Hilus**	2102,7±337	3225,5±104	4367±281	5807,8±273
**Dentate Gyrus**	2305±440	1529±209	3487,9±339,8	4725±520
**NPY-IR cells**				
**CA1 stratum oriens**	781,7±105	734,43±57,9	1145,95±245	2279,48±265,6
**CA1 pyramidal layer**	927,3±148,49	1211,02±116,16	1686,296±230,74	3294,14±205,3
**Hilus**	1930,8±268,19	2870,18±202,06	3043,56±328,78	4139,32±348
**PV-IR cells**				
**CA1 stratum oriens**	2055,5±124,8	2376,1±165	2540,9±237,5	3033,1±114,3
**CA1 pyramidal layer**	2517,2±401,8	2884,6±384	3332,5±296,5	3986,7±459
**CA3 stratum oriens**	501,6±50,9	612,5±60	736,2±125,2	896,1±97,4
**CA3 pyramidal layer**	2130,5±220,4	2414,5±158	2417,8±258,9	3158,2±276,2
**Dentate Gyrus**	1354,7±181,5	1501,1±175	1195,6±193	1554,5±216,9

Supplementary Figure 1: Coexpression of the interneuronal markers neuropeptide Y (NPY) and glutamic acid decarboxylase 67 (GAD67) in the CA1 and hilus hippocampal regions of the different experimental groups.

Representative confocal microscopy micrographs of hippocampal sagittal sections from CA1 (A-D) and CA3 (E-H) hippocampal regions of CTRL+oil- (A, E), CTRL+E2- (B, F), TMT+oil- (C, G), TMT+E2- (D, H) treated rats double-labeled for GAD67 (red) and NPY (green). Scale bar: 40 μm in A-D; 80 μm in E-H. I: Bar graphs indicating the percentage of NPY/GAD67 double-stained cells in CA1 stratum oriens, CA1 pyramidal layer and hilus. The values are given as mean ±SE (* $p < 0.05$, ** $p < 0.001$).

No differences in the percentage of GAD67/NPY double-stained cells are detectable in the various experimental groups (A-I).
