# Supplementary figures and images for: Estrogen administration modulates hippocampal GABAergic subpopulations in the hippocampus of trimethyltin-treated rats
Source: Front Cell Neurosci. 2015 Nov 5;9:433. doi: 10.3389/fncel.2015.00433 (PMC4633568; doi:10.3389/fncel.2015.00433)

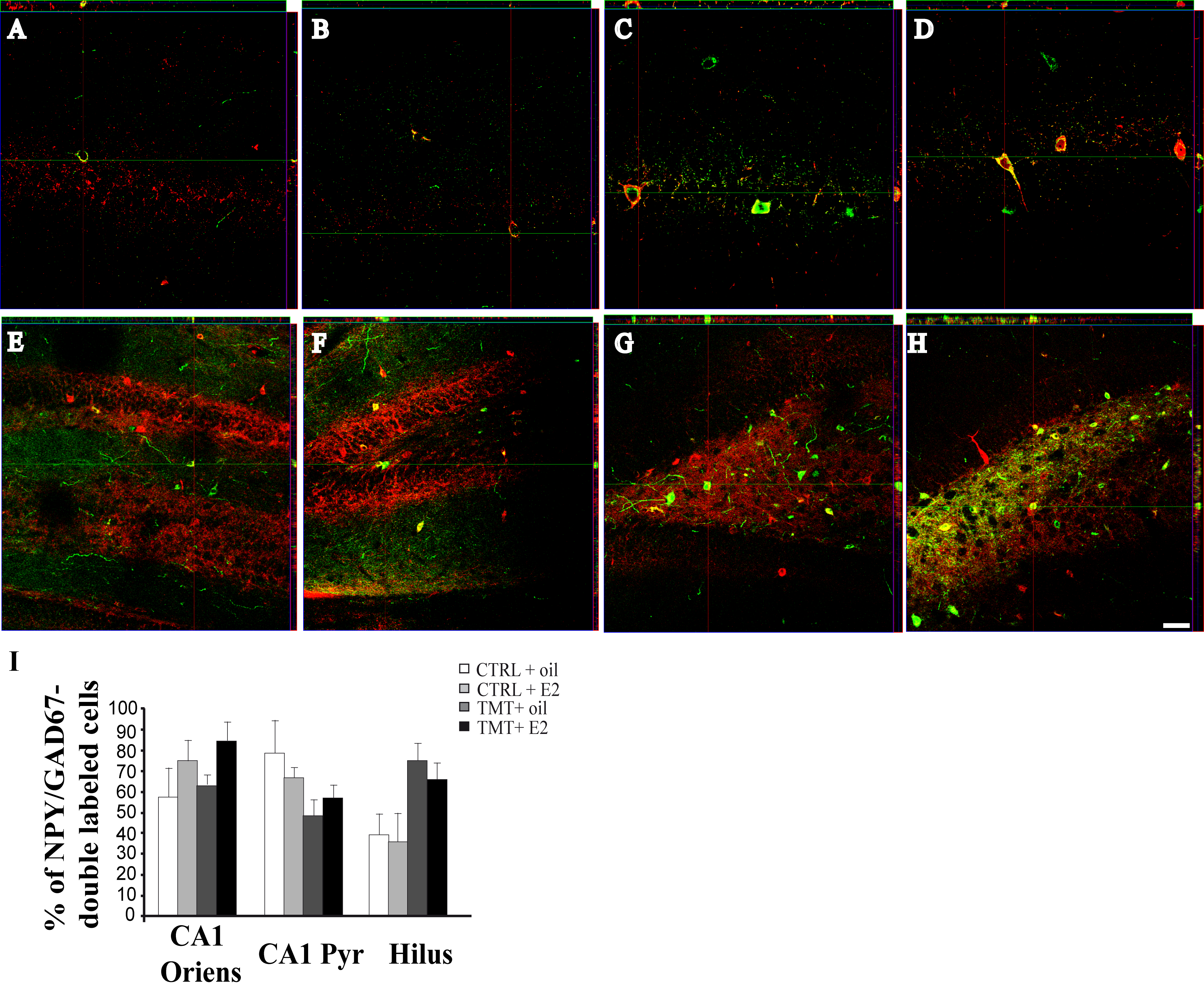

Supplement: Supplementary file 2 [file Image_1.TIF]
